# Supplementary figures and images for: Assessment of Nit-Occlud atrial septal defect occluder device healing process using micro-computed tomography imaging
Source: PLoS One. 2023 Apr 24;18(4):e0284471. doi: 10.1371/journal.pone.0284471 (PMC10124873; doi:10.1371/journal.pone.0284471)

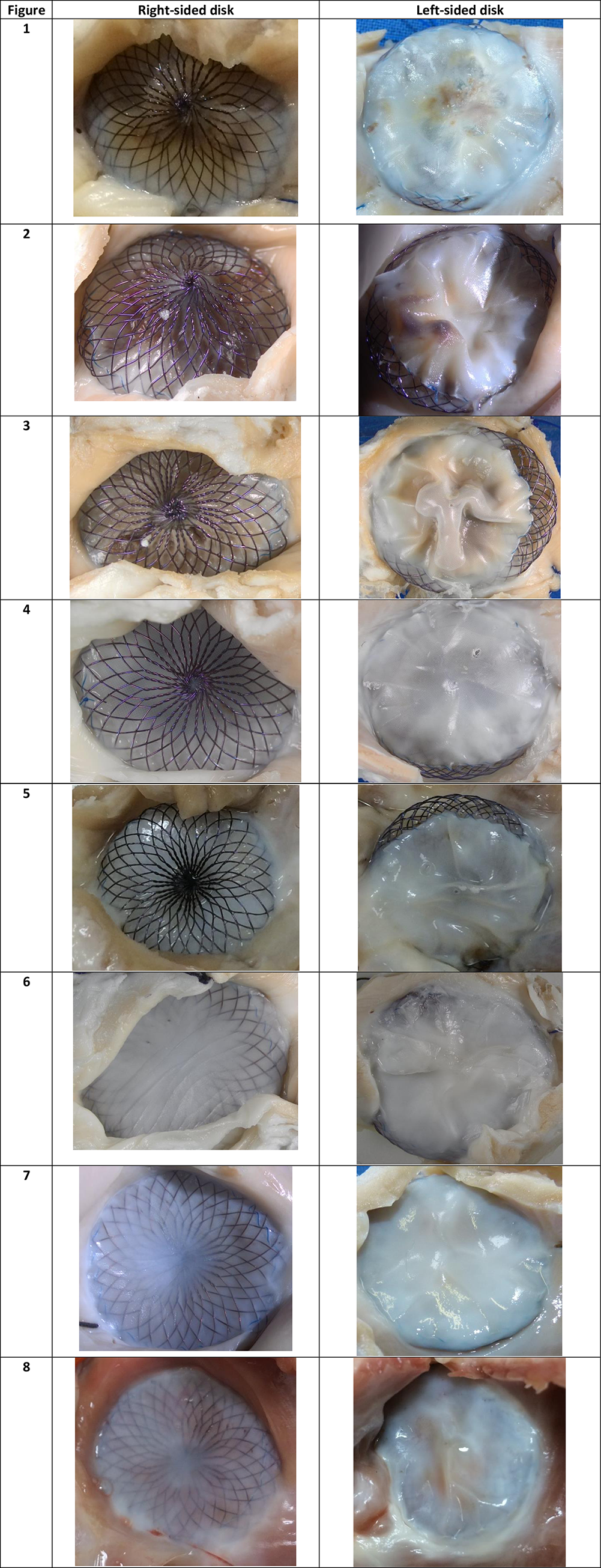

Supplement: S2 File — (TIF) [file pone.0284471.s002.tif]
