# Supplementary material for: Assessment of Nit-Occlud atrial septal defect occluder device healing process using micro-computed tomography imaging
Source: PLoS One. 2023 Apr 24;18(4):e0284471. doi: 10.1371/journal.pone.0284471 (PMC10124873; doi:10.1371/journal.pone.0284471)
Supplement: Attachment — Submitted filename: Response to reviewers.docx [file pone.0284471.s003.docx]

**RESPONSE TO REVIEWERS**

Journal Requirements:

When submitting your revision, we need you to address these additional requirements.

1. Please ensure that your manuscript meets PLOS ONE's style requirements, including those for file naming. The PLOS ONE style templates can be found at 
https://journals.plos.org/plosone/s/file?id=wjVg/PLOSOne_formatting_sample_main_body.pdf and 
https://journals.plos.org/plosone/s/file?id=ba62/PLOSOne_formatting_sample_title_authors_affiliations.pdf

*=> We have taken into consideration this remark.*

2. To comply with PLOS ONE submissions requirements, in your Methods section, please provide additional information regarding the experiments involving animals and ensure you have included details on all efforts to alleviate suffering.

*=> We provided additional information regarding the experiments involving animals and ensured to have included details on all efforts to alleviate suffering.*

3. We note that Figure 1 in your submission contain copyrighted images. All PLOS content is published under the Creative Commons Attribution License (CC BY 4.0), which means that the manuscript, images, and Supporting Information files will be freely available online, and any third party is permitted to access, download, copy, distribute, and use these materials in any way, even commercially, with proper attribution. For more information, see our copyright guidelines: http://journals.plos.org/plosone/s/licenses-and-copyright.
 
We require you to either (1) present written permission from the copyright holder to publish these figures specifically under the CC BY 4.0 license, or (2) remove the figures from your submission:
 
a. You may seek permission from the original copyright holder of Figure 1 to publish the content specifically under the CC BY 4.0 license. 
 
We recommend that you contact the original copyright holder with the Content Permission Form (http://journals.plos.org/plosone/s/file?id=7c09/content-permission-form.pdf) and the following text:
“I request permission for the open-access journal PLOS ONE to publish XXX under the Creative Commons Attribution License (CCAL) CC BY 4.0 (http://creativecommons.org/licenses/by/4.0/). Please be aware that this license allows unrestricted use and distribution, even commercially, by third parties. Please reply and provide explicit written permission to publish XXX under a CC BY license and complete the attached form.”
 
Please upload the completed Content Permission Form or other proof of granted permissions as an ""Other"" file with your submission. 
 
In the figure caption of the copyrighted figure, please include the following text: “Reprinted from [ref] under a CC BY license, with permission from [name of publisher], original copyright [original copyright year].”
 
b. If you are unable to obtain permission from the original copyright holder to publish these figures under the CC BY 4.0 license or if the copyright holder’s requirements are incompatible with the CC BY 4.0 license, please either i) remove the figure or ii) supply a replacement figure that complies with the CC BY 4.0 license. Please check copyright information on all replacement figures and update the figure caption with source information. If applicable, please specify in the figure caption text when a figure is similar but not identical to the original image and is therefore for illustrative purposes only.

*=> We decided to remove the figure under copyright from the submission.*

Additional Editor Comments:
Please response to the reviewers point by point

[Note: HTML markup is below. Please do not edit.]

Reviewers' comments:

Reviewer's Responses to Questions

**Comments to the Author**

1. Is the manuscript technically sound, and do the data support the conclusions?

The manuscript must describe a technically sound piece of scientific research with data that supports the conclusions. Experiments must have been conducted rigorously, with appropriate controls, replication, and sample sizes. The conclusions must be drawn appropriately based on the data presented.

Reviewer #1: Yes

Reviewer #2: Yes

2. Has the statistical analysis been performed appropriately and rigorously?

Reviewer #1: N/A

Reviewer #2: I Don't Know

3. Have the authors made all data underlying the findings in their manuscript fully available?

The [PLOS Data policy](http://www.plosone.org/static/policies.action#sharing) requires authors to make all data underlying the findings described in their manuscript fully available without restriction, with rare exception (please refer to the Data Availability Statement in the manuscript PDF file). The data should be provided as part of the manuscript or its supporting information, or deposited to a public repository. For example, in addition to summary statistics, the data points behind means, medians and variance measures should be available. If there are restrictions on publicly sharing data—e.g. participant privacy or use of data from a third party—those must be specified.

Reviewer #1: No

Reviewer #2: Yes

4. Is the manuscript presented in an intelligible fashion and written in standard English?

PLOS ONE does not copyedit accepted manuscripts, so the language in submitted articles must be clear, correct, and unambiguous. Any typographical or grammatical errors should be corrected at revision, so please note any specific errors here.

Reviewer #1: Yes

Reviewer #2: Yes

5. Review Comments to the Author

Please use the space provided to explain your answers to the questions above. You may also include additional comments for the author, including concerns about dual publication, research ethics, or publication ethics. (Please upload your review as an attachment if it exceeds 20,000 characters)

Reviewer #1: This article by Dr. Perdreau and colleagues from Bordeaux University Hospital reported the assessment of healing process of implanted ASD devices using micro-CT and histological analyses. In general, the idea is unique and interesting. These data are also valuable and important for all the scientists and physicians working on ASD device development.

For better understanding of healing process, immunohistochemical staining of ET cells would be necessary.

I have several comments and suggestions.

1. In abstract, and in line 264-265, clarify the follow up periods in the data of surface coverage of 93 % for LA side and 55% for RA side. Are these data from all 8 animals?
As there are two groups with different follow up periods, it should be separately described.

*=> The median of surface coverage was calculated for the 8 cases. We also provided the calculation for the two groups.*

2. Describe the possible reason why LA side had higher coverage in discussion.

*=> The complete deployment of the occluder showed improved coverage but also a higher propensity for total transoccluder tissue replacement. The mechanisms promoting elevated tissue replacement remains to be investigated. These may include the mechanical status of the surrounding tissue, smaller occlusion diameters and reduced transoccluder distances and the efficiency of septal occlusion.*

3. Histological analysis should include endothelial cells specific staining.

*=>The goal of histology was to identify the device coverage. Richardson blue staining allows for definite identification of endothelial cells based on morphological criteria. Thus, we decided not to perform endothelial cells specific staining. Here is a highly zoomed image of the histological analysis where we can recognize endothelial cells (outer layer at the superior part of the picture and around the metal struts).*

4. There was no figure legends. Provide it.

*=> As recommended by the submission guidelines, we provided the figure legends directly in the manuscript.*

5. Describe the success rate of Brockenbrough technique and if there was any support image (like TEE or ICE) for this?

*=> The success rate of Brockenbrough technique was 100%. We added a TEE image to support it.*

Reviewer #2: The title does not meet study design/ composition. Micro CT was used. It should be mentioned. Also only ASD device: Nit occlud was studied. Results should not generalised for all devices.

*=*> *We changed the title, taking into consideration your recommendations.*

Altough the aim of the study is to evaluate device coverage process, the feasibility of CT scan for this purpose, authors studied this only after heart explant. The CT scans performed before heart explant and a second CT scan after explantation process should be beneficial for comparing the results of images with beating and non-beating heart. If the aim was evaluting the epitalization status CT scan is not necessary we can see macroscopically and microscopically. If they want to evalute the feasibility of CT scan than it was necessary to evalute it on beating heart.

We know that when we make a cardiac scan with CT on beating heart with high rates results has many image artefacts. Was that the fact that they perform the study after explantation?

*=> We are addressing a need to better evaluate the coverage of devices in a preclinical setting. We found that micro-CT robustly characterizes the full 3D aspect of the device and its coverage, that no other imaging tool can effectively achieve in the same time frame. A beating heart system would aid understanding how this type of evaluation would perform in clinical applications, but the tools and resolution on the systems are not currently available. Despite this, CT and micro-CT systems are capable of ECG-gated imaging acquisition. Therefore, the potential for beating heart imaging is already evident for future studies and clinical applications.*

So what is the clinical and practical advantage of this study? Discussion must focus on this subject.

*=> Our study provides the resolution requirements of CT imaging to characterize tissue coverage of the occluders. Developments in clinical imaging systems remain to be put in place to meet these requirements for application in patient care. However, the study provides a methodology for preclinical evaluation and robust device testing. The determinants for successful tissue coverage are namely complete deployment of the occluder. This study highlights the main factors to verify during procedures to improve treatment occlusion efficiency. We included this question in the discussion of the manuscript.*

6. PLOS authors have the option to publish the peer review history of their article ([what does this mean?](https://journals.plos.org/plosone/s/editorial-and-peer-review-process#loc-peer-review-history)). If published, this will include your full peer review and any attached files.


If you choose “no”, your identity will remain anonymous but your review may still be made public.


**Do you want your identity to be public for this peer review?** For information about this choice, including consent withdrawal, please see our [Privacy Policy](https://www.plos.org/privacy-policy).

Reviewer #1: No

Reviewer #2: No
