# Supplementary material for: Assessment of Nit-Occlud atrial septal defect occluder device healing process using micro-computed tomography imaging
Source: PLoS One. 2023 Apr 24;18(4):e0284471. doi: 10.1371/journal.pone.0284471 (PMC10124873; doi:10.1371/journal.pone.0284471)
Supplement: Attachment — Submitted filename: PLOS ONE revision .docx [file pone.0284471.s004.docx]

First, I would like to congratulate Dr Perdreau et al et al for their work in using a sheep model and trying to evaluate its endothelialization process with Micro CT. It is hoped that such minimally invasive imaging evaluation methods will be applied clinically in the future.

I have a few questions and points to make.

1) Why did this group use the sheep model instead of pigs, etc.? An explanation is needed.

2) Why did you use PFM medical out of all the ASD devices?　Explanation is needed.

3) Did you acquire ICE or TEE echo-images during the surgery?　When experimenting with ASD devices in sheep, it should be impossible to place them in the correct position without ICE or other guiding.　And even with echo, you need to follow up to see if the shunt, etc. is closed and the implanted device is in the correct position.

4) Dr Perdreau et al. have animals sacrificed at 1 month and 3 months, but the observation period is too short. If endothelialization is to be considered, shouldn't evaluation at 6 months and 1 year, etc. be necessary?

5) If endothelialization is to be evaluated, immunostaining with CD 31,vWF seems necessary.

6) Why is endothelialization on the right atrial side slower than on the left atrial side? This should be added to the discussion.
