# Supplementary material for: Assessment of Nit-Occlud atrial septal defect occluder device healing process using micro-computed tomography imaging
Source: PLoS One. 2023 Apr 24;18(4):e0284471. doi: 10.1371/journal.pone.0284471 (PMC10124873; doi:10.1371/journal.pone.0284471)
Supplement: Attachment — Submitted filename: Response to reviewers.docx [file pone.0284471.s005.docx]

**RESPONSE TO REVIEWERS**

Reviewer #1: I think that the authors appropriately answered all of the questions by the reviewers. And the revision was appropriately done and the revised manuscript is much improved.

*We thank this reviewer for the constructive comments and revision.*

Reviewer #3: First, I would like to congratulate Dr Perdreau et al et al for their work in using a sheep model and trying to evaluate its endothelialization process with Micro CT. It is hoped that such minimally invasive imaging evaluation methods will be applied clinically in the future.
I have a few questions and points to make.

*We thank you for reviewing our work. We carefully answered the questions (see below).*

1) Why did this group use the sheep model instead of pigs, etc.? An explanation is needed.

*We chose a sheep model instead of pig model because a part of the inital experimental protocol included MRI, so we needed an animal that was not going to put on weight too much. The healing process was also close to human being.*

2) Why did you use PFM medical out of all the ASD devices? Explanation is needed.

*A reduced metal content and absence of protruding fixation clamps improved the compatibility of the Nit-Occlud ASD-R occluder with computed tomography-based imaging techniques, which are prone to artefacts associated with over attenuation of X-rays. Before this experimental protocol, we notably experimented the use of Amplatzer septal occluder but the protruding fixation resulted in important artefacts when performing CT scan.*

3) Did you acquire ICE or TEE echo-images during the surgery? When experimenting with ASD devices in sheep, it should be impossible to place them in the correct position without ICE or other guiding. And even with echo, you need to follow up to see if the shunt, etc. is closed and the implanted device is in the correct position.

*We used TTE to guide the procedure (with fluoroscpic guidance associated) and acquired echo-images during the procedure : TTE allowed to follow the wire to perform trans-septal puncture, see the absence of atrial shunt at the end of the procedure and the correct position of the device. We included an example of acquired echo-image in Figure 1. Before sacrifice, we performed TTE to check the absence of residual shunt and confirm good position of the device. We manage to obtain images of enough quality and didn’t need to use TEE.*

4) Dr Perdreau et al. have animals sacrificed at 1 month and 3 months, but the observation period is too short. If endothelialization is to be considered, shouldn't evaluation at 6 months and 1 year, etc. be necessary?

*We chose this period of observation, regarding the duration found in literature (28-90 days), which is quicker than in humans. We can also see that endothelialization of the device was complete after 3 months for devices without any protrusion of the disk. Having incomplete endothelialization of some devices allowed to be sure to identify the covering tissue.*

5) If endothelialization is to be evaluated, immunostaining with CD 31,vWF seems necessary.

*The goal of histology was to identify the device coverage. Richardson blue staining allows for definite identification of endothelial cells based on morphological criteria. Thus, we decided not to perform endothelial cells specific staining. Here is a highly zoomed image of the histological analysis where we can recognize endothelial cells (outer layer at the superior part of the picture and around the metal struts).*

6) Why is endothelialization on the right atrial side slower than on the left atrial side? This should be added to the discussion.

*Disk conformation appears to determine, in part, device coverage. The complete deployment of the occluder showed improved coverage but also a higher propensity for total transoccluder tissue replacement. Complete transoccluder tissue replacement in fully deployed occluders appeared to facilitate tissue coverage of the right disk. The mechanisms promoting elevated tissue replacement remains to be investigated. These may include the mechanical status of the surrounding tissue, smaller occlusion diameters, reduced transoccluder distances and the efficiency of septal occlusion. This was added in the discussion.*
